# Effect on Intermediary Metabolism and Digestive Parameters of the High Substitution of Fishmeal with Insect Meal in *Sparus aurata* Feed

**DOI:** 10.3390/insects12110965

**Published:** 2021-10-25

**Authors:** Dmitri Fabrikov, María del Carmen Vargas-García, Fernando G. Barroso, María José Sánchez-Muros, Sylvia María Cacua Ortíz, Amalia E. Morales, Gabriel Cardenete, Cristina Tomás-Almenar, Federico Melenchón

**Affiliations:** 1 CEIMar, CECOUAL-Department, Biology and Geology, University of Almería, 04120 Almería, Spain; df091@ual.es (D.F.); fbarroso@ual.es (F.G.B.); mjmuros@ual.es (M.J.S.-M.); 2 GDCON Group, Environmental School, Faculty of Engineering, University of Antioquia, Medellín 050010, Colombia; sylvia.cacua@udea.edu.co; 3 Department Zoology, Facultad de Ciencias, Campus Fuentenueva, University of Granada, 18071 Granada, Spain; amaenca@ugr.es (A.E.M.); gcardenete@ugr.es (G.C.); 4 Agro-Technological Institute of Castilla y León, Ctra. Arévalo, s/n, 40196 Segovia, Spain; critoal@gmail.com (C.T.-A.); melramfe@itacyl.es (F.M.)

**Keywords:** aquafeed, *Sparus aurata*, insect meal, *Tenebrio molitor*, *Hermetia illucens*, biometric parameters, gut microbiome

## Abstract

**Simple Summary:**

The depletion of traditional protein sources and the impact this causes on the production costs of aquaculture feed make it necessary to find alternative materials that allow for the sustainability of production. Among various proposals, insects have drawn scholarly attention because of their high protein content and the efficiency of their production, both from an environmental and an economic perspective. However, nutritional changes in fish diets require further clarification regarding the effect of this new ingredient in fish performance and physiology. In this study, we evaluated the use of two insect meal species, *Hermetia illucens* and *Tenebrio molitor*, for the partial replacement of fishmeal, as well as their influence on growth indices and the gut microbiome. Although the results showed a worsening of biometric parameters and a modification of the microbial community, the impact was different depending on the insect species and their rearing conditions. Thus, specific studies for each case are recommended.

**Abstract:**

*Hermetia illucens* and *Tenebrio molitor* were tested on account of their potential to replace fish protein in feed. Two levels of replacement for *H. illucens*, 30% and 50% (H30 and H50), and one for *T. molitor*, 50% (T50), as well as an additional diet with a modified fatty acid fraction (H50M), were investigated in relation to juvenile *Sparus aurata* growth indices, enzyme activities and gut microbiome. A T50 diet showed similar results to a control (C) diet, with no significant differences regarding morphological indices and minor differences for nutritional indices. Regarding the gut microbiome, H50M was the diet which showed the more similar prokaryotic community to C, which suggests that fatty acid fractions might influence the composition of the gut microbiome. Nevertheless, differences appeared to be related to a redistribution of dominant species, while changes in species affiliation were limited to minoritary species. The positive correlation between some of these minoritary species (*Peptostreptococcus russellii*, *Streptococcus dysgalactiae* and *Weisella confusa*) and several fish growth parameters might explain differences between control and insect diets. Deciphering such uncertainty and revealing the potential role these unusual species may play on fish performance should be addressed in future investigations.

## 1. Introduction

A circular economy has become a target objective in the movement towards sustainability. Insects play an important role in this change because their rearing has a small ecological footprint; less land and water is required, they emit fewer greenhouse gases, and they have a high feed conversion efficiency in comparison to traditional animal products. These factors should be considered in tandem with insects’ capacity to transform low-value organic by-products into high-quality food or feed that can be used as animal feed or aqua feed [1].

The utilization of insect meal as a substitute in fishmeal has been studied in several fish species and insect species. High percentages of substitution have been used for feeding several species without compromising their growth ranges, as in the case of *Salmo salar* [2], yellow catfish [3] and Nile tilapia [4], with substitution levels of 100%, 75% and 68%, respectively. However, most of the studies reported 25% as the threshold at which growth indices can be negatively affected [5]. Among the most studied species are *Hermetia illucens* (HI) and *Tenebrio molitor* (TM), which, in addition to meeting the approval of the EU as feed ingredients, are produced in sufficient quantities to sustain animal feed manufacturing.

The effect of the inclusion of HI in aqua feed has been analyzed in relation to many fish species and many different issues, such as disease resistance, innate immune response, growth performance, intestinal antioxidant enzymes and amino acid composition [6]. Similar studies have been carried out for TM [7,8].

*Sparus aurata*, gilthead seabream, is a widely consumed fish in the Mediterranean area. The culture of *Sparus aurata* is expanding, as evidenced by 96% of its total production (aquaculture + fisheries) corresponding to aquaculture in 2016. In the period of 2012–2016, cultivated production increased by 32%, and an additional 14% from 2016 to 2017, reaching 94,936 tons and EUR 485 million [9]; this was mostly fueled by an increase in production in the Mediterranean Sea [9]. Currently, feeds for seabream are based in fishmeal and soy as protein sources. Nevertheless, sources of soy and fishmeal present environmental problems. To reduce their drawbacks, insect meal can be considered as a potential substitute due to its more sustainable nature and broad acceptance [10].

The inclusion of both insect species, HI and TM, in *S. aurata* feed has been studied previously [11]. Results pointed out the existence of a limit of substitution, 25–30%, above which growth indices worsened in comparison with fishmeal diets, although two recent studies by Pulido-Rodriguez et al. [12] and Randazzo et al. [13] reported similar or even better results with a level of substitution of 40% of vegetable protein mixed with HI meal. Nevertheless, higher percentages of substitution have been reported using other alternative protein sources, such as soy (40%) [14] or pea protein concentrates (60%) [15]. One of the main reasons for this limit of insect inclusion is attributed to the low digestibility of chitin and scleroprotein of insect exoskeletons [10]. Another handicap that limits the inclusion of insects in fish diets is the fatty acid profile, which is different from fishmeal. Insects have a higher percentage of n-6 polyunsaturated fatty acids (*n*-6 PUFAs), whereas fishmeal is richer in n-3 highly unsaturated fatty acids (*n*-3 HUFAs). In this sense, and trying to overcome this last challenge, Fabrikov et al. [16] obtained *n*-3 HUFAs-rich larvae feeding HI in discards from fisheries, and Liland et al. [17] produced similar results by feeding larvae with seaweed-enriched media.

Despite the performance of insect meal in relation to fish acceptance or its impact on growth parameters, the effects of high levels of inclusion on digestive and metabolic processes are not well known, and more knowledge is needed to realize the barriers precluding the use of insect meal in aquafeed. In this sense, it is also important to elucidate modifications in the gut microbiome due to the inclusion of insect meal in fish diets. Microbial communities associated with the intestinal tract play a decisive role not only in relation to nutrient assimilation, but regarding immunological responses and disease prevention [18]. Among these communities, some are considered as persistent and others are understood as transient [19], depending on their temporality. The presence of transient microbiota is subject to fluctuations caused by environmental factors, whereas persistent communities are less affected by such conditions and only minor quantitative modifications can be observed. Among the most influential factors regarding the composition of transient microbiota are those of a nutritional nature [20]; these can promote the temporary presence of species more adapted to the properties of substrates used and, consequently, can foster the better utilization of a diet as a whole [21]. Thus, the study of the gut microbiome and its response to changes in the nutritional composition of feeds can help to interpret the specific roles of different species and even select potential probiotics adapted to the type of nutrients being used. Ultimately, this understanding might encourage the better use of alternative protein sources and allow for the partial and even full replacement of traditional fishmeal-based diets.

Considering the abovementioned discussion, the main aim of this study was to investigate the effect of high levels of replacement of fishmeal with TM or HI meal—including meal with a modified acid profile—on growth and nutritive indices, digestive and metabolic enzymes and the gut microbiota of *S. aurata* fed with such diets. Elucidating potential correlations between growth parameters and microbial communities was also established as a primary goal.

## 2. Materials and Methods

### 2.1. Insect Meals

HI and TM larvae used in this experiment were purchased from Entomotech S.L. and MealFood Europe S.L., respectively. One group of HI had been fed with fishery discards obtained from the port of Almería (Spain) to increase their nutritional value. Insects were dried and ground to obtain full-fat insect meal. Table 1 and Table 2 show the proximal composition and aminoacidic profiles of the two HI and TM larvae, respectively. Methodologies applied for the determination of analyzed parameters are described in Section 2.4.1.

### 2.2. Diets

Five diets were prepared using different substitution levels of fishmeal by insect meal: the control diet (C) was prepared with fishmeal only, 30% substitution of fishmeal with HI meal (H30), 50% substitution of fishmeal with HI meal (H50), 50% substitution of fishmeal with fish-fed HI meal (H50M) and 50% substitution of fishmeal with TM meal (T50). For the H50M diet, HI larvae were fed with fish discards to modify their fatty acid profile by increasing their polyunsaturated fatty acid content. The formulated diets were isoenergetic, isoproteic and isolipidic. Table 3 shows the ingredients and proximal composition of the formulated diets.

### 2.3. Fish Feeding Trial and Sampling

Juvenile *Sparus aurata*, with an average weight of 7.10 g (Wi, initial weight), were obtained from Predomar S.L. (Carboneras, Spain) and reared during the experimental period in the Aquarium of University of Almería (Almería, Spain). Fish were adapted to aquarium conditions over 15 days under constant temperature (20 ± 1 °C) and a natural photoperiod. After the acclimatation period, 450 fish were individually weighed and randomly distributed in 15 300 L experimental tanks (3 tanks/treatment; 30 fish/tank). For each treatment, three 300 L tanks were used. Fish were fed ad libitum twice a day at 09:00 and 14:00. Feed intake was recorded daily, and the experiment ended when fish fed on C diet tripled their body weight (32 days).

Before the end of the experiment, fish were fasted for 24 h prior to sampling. Fish were anaesthetized and weighed. For sample collection, 6 fish/tank were sacrificed with an overdose of anesthesia (clove oil, 100 mg/L); then, viscera were extracted and weighed. The liver, intestine, stomach and muscle were separated, weighed, frozen in liquid N_2_ and stored at −80 °C until further analysis. To analyze apparent digestibility, feces samples were collected during the last two weeks of the growth trial by a modified Guelph method [22], gathering the feces produced throughout 24 h in a settling column. The feces samples were frozen at −80 °C until further analysis.

### 2.4. Sample Analysis 

#### 2.4.1. Proximal Composition 

The nutritional profiles of insect meals, diets and fish used in all experiments were analyzed according to the standard procedures of the Association of Official Analytical Chemists [23], with specific methods as follows: crude protein content was determined by Kjeldahl (AOAC, 2005; #954.01) using a conversion factor of 6.25 for feeds, feces and muscle, and 4.67 for HI and 4.75 for TM; crude fat (EE) was determined by ethyl ether extraction (Soxhlet technique) (AOAC, 2005; #920.39); the moisture was gravimetrically quantified by drying at 105 ± 0.5 °C (AOAC, 2005; #934.01), whereas ash was gravimetrically determined after combustion at 500 °C in a muffle furnace (AOAC, 2005; #942.05) to a constant weight. Phosphorus quantification was achieved according to protocol ISO standard (1996) (ISO 13730) based on molecular absorption spectrophotometry (UV/Vis UV2, UNICAM, Cambridge, UK). Calcium was determined following the protocol described by Pessoa (2016) using the X-ray fluorescence method of dispersive energy (ED-XRF). Gamage and Shahidi’s protocol (2007) was used for the obtention of chitin residue, which was washed with acetone, dried, and weighed. For the obtention of the amino acid profiles, hydrolysates from insect meal samples were obtained by mixing 1.5 mg with 200 µL of 6 N HCl for 22 h at 110 °C; these were then qualitatively and quantitatively analyzed by ion-exchange liquid chromatography and post-column continuous reactions with ninhydrin (Biochrom 30 AAA series, Biochrom, UK). The apparent digestibility of the protein was determined using acid-insoluble ash as a marker in feeds and feces [24].

#### 2.4.2. Digestive Enzymes

Individual intestines and stomachs were homogenized in distilled water (1:1, *w*/*v*) with Polytron PT 2100 (Kinematica AG Inc., Lucerne, Switzerland) at 15,000 rpm in an ice bath. Extracts were centrifuged at 20,000× *g* (Orto Alresa Biocen 22r) at 4 °C for 20 min. Protein concentrations of extracts were measured with a Pierce™ BCA Protein Assay Kit (Thermo Scientific TM, Rockford, IL, USA) to obtain appropriate concentrations of enzymes for activity analysis. Acid protease activity was measured in stomach extract following the method described by Anson [25], and 0.5% hemoglobin was used as a substrate. Alkaline protease and alpha-amylase activities were measured from intestine extract. Alkaline protease activity was measured according to the method described by Walter [26], and alpha-amylase activity was measured following the method described by Somogyi-Nelson [27]; 1% casein and 1% starch were used as substrates, respectively. Absorbance was measured at 280 nm for protease and 560 nm for alpha-amylase using a Power Wavex microplate scanning spectrophotometer (Bio-Tek Instruments, Winooski, VT, USA) in duplicate in 96-well microplates (UV-Star Greiner Bio-One, Frickenhausen, Germany). Protease activity was described as the micrograms of tyrosine produced per minute and per milligram of protein. Alpha-amylase activity was described as the micrograms of maltose produced per minute and per milligram of protein at 37 °C.

#### 2.4.3. Metabolic Enzymes

Individual livers were homogenized (1:9, *w*/*v*) in buffer solution (Tris/HCl 0.1 M, Triton X-100 0.1%, EDTA 0.25 mM and NaCl 0.01 M pH 7.6) with Polytron PT 2100 at 15,000 rpm in an ice bath. The mixture was centrifuged at 20,000× *g* for 30 min at 4 °C. Protein concentrations of extracts were measured with a Pierce™ BCA Protein Assay Kit (Thermo Scientific TM, Rockford, IL, USA) to obtain appropriate concentrations for each enzyme activity analysis.

Alanine aminotransferase (EC 2.6.1.2, ALT) was measured using the method described by Lain-Guelbenzu et al. [28] with L-alanine as a substrate. Aspartate aminotransferase (EC 2.6.1.1, GOT) was measured using the method described by Krista and Fonda [29] with L-aspartate as a substrate. Glutamate dehydrogenase (EC 1.4.1.2, GDH) was measured using the method described by Katoh et al. [30] with α-ketoglutarate as substrate. Pyruvate kinase (EC 2.7.1.40, PK), glucose 6-phosphate dehydrogenase (EC 1.1.1.49, G6PDH) and fructose 1,6-bisphosphatase (EC 3.1.3.11, FPBase) were measured according to methods described by Furné et al. [31], using phosphonyl pyruvate, D-glucose 6-phosphate and D-fructose 1,6-biphosphate as substrates, respectively. Absorbance was measured at 340 nm using a Power Wavex microplate scanning spectrophotometer in duplicate in 96-well microplates. Activity of the enzyme was expressed as the amount of enzyme required to oxidize/reduce 1 µmol of NADH/NADP per minute and protein at 25 °C.

#### 2.4.4. Gut Microbiota

Sequencing and bioinformatics were performed by Life Sequencing S.A. (Valencia, Spain). Gastrointestinal tract samples were processed in order to achieve the DNA extraction using the QiAamp power fecal Pro DNA kit (Qiagen Sciences, Germantown, MD, USA), according to the protocol described by Lyons et al. [32]. DNA extracts were tested for concentration and quality using a NanoDropTM 3300 spectrophotometer (Thermo Fisher Scientific, Waltham, MA, USA) and used to construct the corresponding genomic libraries from the V3-V4 hypervariable region 16s rRNA gene [33], applying universal primers S-D-Bact-0341—b-S-17 (5′-CCTACGGGNGGCWGCAG-3′) and S-D-Bact0785-a-A-21 (5′-GACTACHVGGGTATCTAATCC-3′). Sequencing analysis was carried out on an Illumina MiSeq platform (Illumina, Ic., San Diego, CA, USA) utilizing a 300 bp fragments paired-end protocol. The amplification cycle was as follows: initial denaturation at 95 °C for 5 min, 25 cycles consisting of denaturation at 95 °C for 40 s, annealing at 55 °C for 2 min, extension at 72 °C for 1 min and a final extension step at 72 °C for 7 min. PEAR software v.0.9.1 (http://cme.h-its.org/exelixis/web/software/pear, accessed on 21 May 2021) was applied for the construction of the overlapped sequences and CUTADAPT software v.1.8.1 for the removal of adapters [34]. Sequences displaying a quality score higher than Q20, length > 200 bp and free-form chimeric sequences by the implementation of Chimera Uchime [35] were selected. The remaining quality sequences were clustered at a 97% cutoff using CD-HITt software v.4.6.8 [36,37] and phylogenetically classified to the maximum taxonomical level possible using the BLAST tool for Local Alignment (NCBI, http://blast.ncbi.nlm.nih.gov/Blast.cgi, accessed on 14 June 2021) and the associated GenBank database.

Shannon–Wiener and Simpson α biodiversity indices were calculated at species taxonomic level using a 97% sequence similarity threshold for the establishment of OTUs. Community similarity among samples (β biodiversity) was estimated by means of the Sørensen–Dice qualitative (presence/absence data) and quantitative (relative abundance data) indices.

### 2.5. Statistical Analysis

Three independent replicates were used in all analyses, and data are presented as the mean ± standard error. A one-way analysis of variance (ANOVA) and a multiple comparison of means based on the Tukey–Kramer HDS approach at a 95% confidence level were performed to test for significant differences between treatments regarding growth and enzymatic parameters. The relationships among all variables (growth, enzymatic and microbiological factors) and the existence of differences between samples were plotted through principal component analysis and correlation analysis. Correlations between variables were determined by Pearson product correlation at both 95% and 99% confidence levels. All statistical treatments were performed using IBM SPSS Statistics v. 26 software (IBM Analytics, Armonk, NY, USA) and Statgraphics Centurion XVI version 18.1.12 (Statpoint Technologies, Inc., Warrenton, VA, USA). 

### 2.6. Ethical Standards

The experiment was conducted according to the directive 2010/63/EU of the European Parliament for the protection of animals used in experiments and for other scientific purposes and was approved by the Órgano Competente of Junta de Andalucía (n° ref.: 19/05/2017/065) according to Royal Decree 53/2013 of 1 February 2013.

## 3. Results

### 3.1. Growth and Nutritive Indices

Table 4 shows nutritional and growth indices worsened by the inclusion of insects, especially in the case of HI diets.

In statistical terms, treatments could be categorized into three groups: control treatment, which showed the best result; the three experimental diets with HI at any percentage of replacement or the use of modified meal; and the T50 diet. Nevertheless, nutritional indices, such as FCE and PER, did not show significant differences among H30, H50M and T50 treatments. On the other hand, and with respect to morphological indices (Table 4), the use of HI meal as an alternative protein source produced significantly lower responses, except for intestine percentage, whereas differences were not found between C and T50 diets. In this case, the results were not always statistically different, because values for H50M were similar to that of C.

Regarding proximal composition of the muscle (Table 5), a similar protein content was found between treatments. A similar pattern was observed in the case of lipid content, although differences were high enough to generate significant differences. Moisture also varied between treatments; lower moisture values were found for the control diet and the highest moisture was observed in the H50M diet. Nevertheless, statistical differences only appeared between control and H30 and H50 diets. Ash contents increased with HI content in the diets, showing statistical differences between the diet using HI and the other diets, except for C and H30.

The apparent digestibility coefficient (ADC) showed lower values for HI-based diets than for C or TM, except for H50M.

### 3.2. Intermediary Metabolism

**Figure 1:**
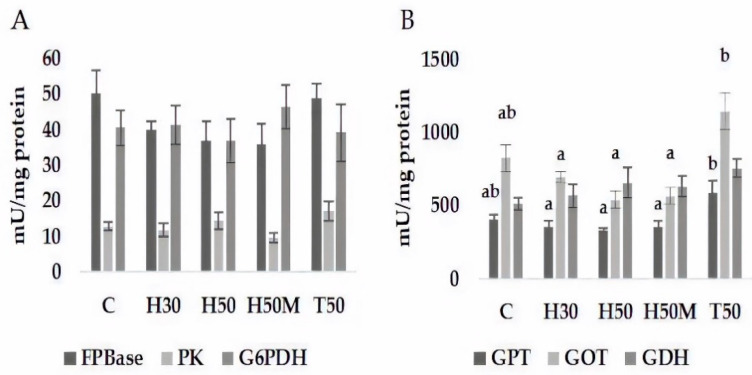
Enzymes of intermediary metabolism of *Sparus aurata* fed with experimental diets. Means (±SE) of three replicate tanks (30 fish/tank). Different letters indicate significant differences (*p* < 0.05) based on Tukey–Kramer HSD tests. Columns without letters indicate an absence of significant differences. Sugar-related enzymes are depicted in Subfigure (**A**) (FPBase (fructose-1,6-biphosphatase); PK (pyruvate kinase); G6PDH (glucose-6-phosphate dehydrogenase)) and amino acid-related enzymes in Subfigure (**B**) (GPT (glutamate pyruvate transaminase); GOT (glutamic oxalacetic transaminase); GDH (glutamate dehydrogenase)).

No significant differences in terms of enzyme activity related to glucose metabolism (Figure 1A) were detected for any of the enzymes determined (PK, pyruvate kinase; FBPase, fructose-1,6-biphosphatase; G6PDH, glucose-6-phosphate dehydrogenase) between different treatments. Regarding enzymes of amino acid metabolism (Figure 1B), results associated with GDH (glutamate dehydrogenase) activity did not give rise to different statistical groups either, whereas those corresponding to GPT (glutamate pyruvate transaminase) and GOT (glutamic oxalacetic transaminase) were divided into two significant groups constituted by, on one hand, HI-based diets, and on the other hand, T50. C treatment remained in an intermediate position, associated with both groups.

### 3.3. Digestive Enzymes

**Figure 2:**
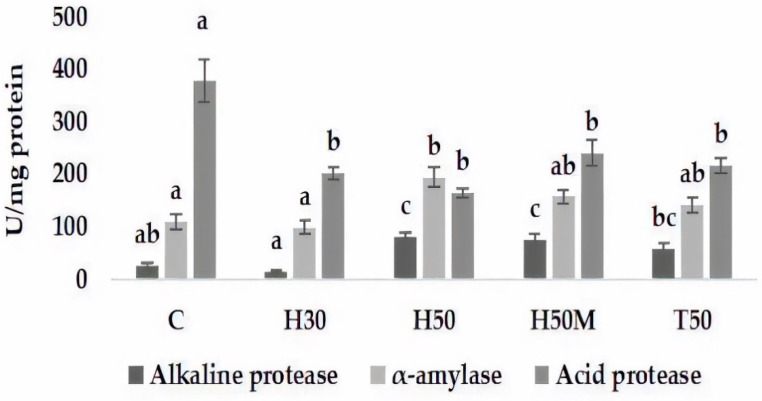
Digestive enzymes of *Sparus aurata* fed with experimental diets. Means (±SE) of three replicate tanks (30 fish/tank). Different letters indicate significant differences (*p* < 0.05) based on Tukey–Kramer HSD tests.

Alpha-amylase and alkaline protease activities (Figure 2) showed similar behavioral patterns, with maximal and minimal levels for H50 and H30 treatments, respectively, and higher values for H50M and T50 in comparison to the C diet.

Regarding acid protease (Figure 2), the inclusion of insect meal had a clear negative effect on the activity of this enzyme, with reductions of around 50%. In contrast, no significant differences were observed between diets including insect meal.

### 3.4. Effect of Insect Meal Inclusion on Bacterial Communities

The inclusion of insect meal in fish diets caused changes in the microbial communities associated with the gastrointestinal tract. Regarding the number of operational taxonomic units (OTUs), such changes showed different trends depending on the insect and the percentage of its inclusion (Table 6). Thus, whereas H30 and T50 promoted an increase in the total number of OTUs, the use of H50 and H50M gave rise to a decrease; this was especially noticeable in the last case. Curiously, a higher number of OTUs did not always correspond to greater diversity expressed by means of the Shannon–Wiener index, because H50M showed the second highest diversity, whereas the minimal value was detected for T50. The values obtained for the Simpson index confirmed this scenario, allocating the lowest level, i.e., greater homogeneity, to T50 samples. It is worth mentioning the case of H30. Curiously, fish fed with this diet showed a more diverse and heterogeneous microbiome than C, but only when the whole community was estimated. In contrast, when only prevalent OTUs were considered (relative abundance > 0.5%) both diversity and heterogeneity were much lower than in the C diet.

Regarding similarities between treatments, expressed by the β Sørensen-Dice diversity index, correspondences between C samples and the rest remained in the 45–50% range (Table 7). From a quantitative perspective, considering the relative abundance of each OTU, the diet that promoted maximal alteration in the composition of the prokaryotic community was H30, whereas H50M preserved the greater percentage of similarity. When comparisons were limited to those OTUs presenting a relative abundance over 0.5% in at least one of the treatments (restricted community), qualitative results increased to levels over 80% in most cases, although quantitative values remained virtually unchanged. Thus, H50M and H30 again showed maximal and minimal quantitative similarities with the C treatment (65% and 27%), values which were nearly identical to those found when the whole population was considered. The highest and lowest qualitative similarity were observed for H30/H50M (97%) and H50/H50M pairs (78%), respectively, whereas such role corresponded to H50/T50 (80%) and H30/C pairs (27%) when quantitative data were analyzed.

**Figure 3:**
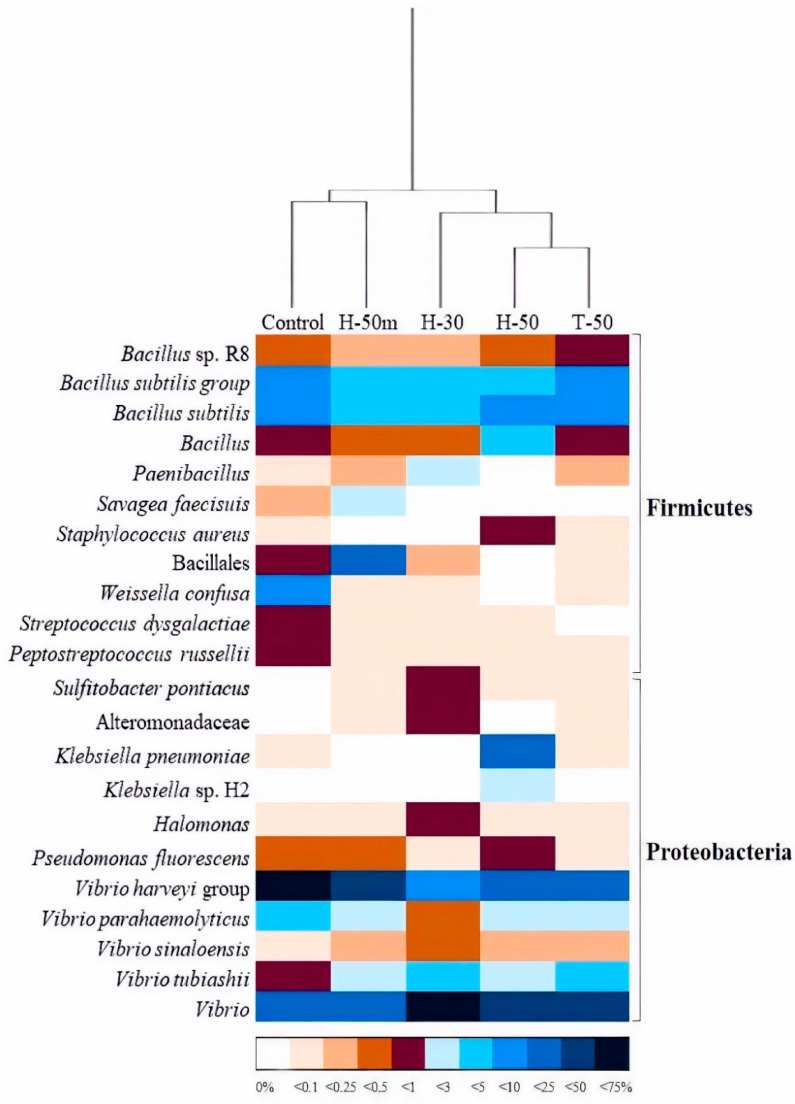
Heatmap representing the distribution, expressed as a percentage, of OTUs within samples and a dendrogram showing the relationships between treatments based on a restricted microbial community.

A more in-depth analysis of this predominant community (relative abundance over 0.5%) (Figure 3) revealed the existence of two main clusters, one composed of C and H50M treatments and the other comprising H30, H50 and T50 samples, with the last two forming a subcluster. All the OTUs found were assigned to Firmicutes and Proteobacteria phyla, with *Bacillus* and *Vibrio* representatives prevailing in all cases. Members of the Bacillales order and the *Vibrio harveyi* group contributed a higher percentage to the C/H50M microbiome cluster, whereas *Vibrio tubiashii* and *Vibrio* sp dominated the prokaryotic community of the H30/H50/T50 cluster. From a global perspective, the inclusion of insect meal, whatever its form, produced a decrease in the relative abundance of *Weissella confusa*, *Streptococcus dysgalactiae* and *Peptostreptococcus russelliii*, as well as the emergence of *Sulfitobacter pontiacus* and representatives of the Alteromonadaceae family.

### 3.5. Interrelationships between Microbial Community and Fish Parameters

**Figure 4:**
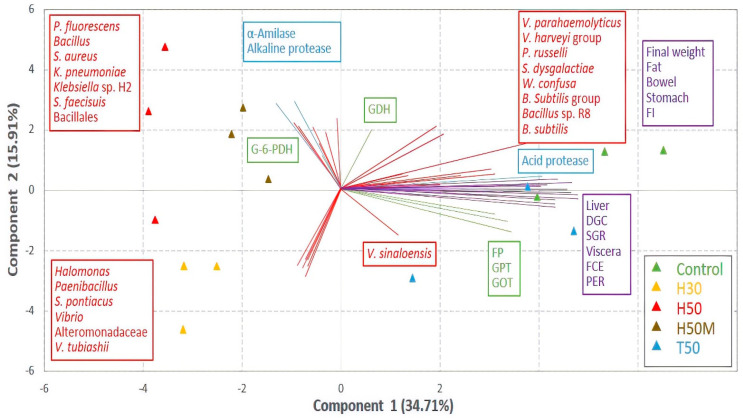
Principal component analysis biplot showing grouping of treatments and relationships amongst variables as influenced by treatments. Metabolic enzymes are depicted in green, digestive enzymes in blue, biometric parameters in violet, and microorganisms in red.

Figure 4 shows results obtained from the principal component and discriminant function analyses. The treatments were divided into two main groups: the first one made up of C and T50 diets, and the second one formed of treatments including HI meal, with a subgroup integrated by H50 and H50M. The first group was related to all biometric parameters and most of the metabolic enzymes, whereas the associated bacterial community was mainly dominated by Gram-positive species. On the contrary, the parameters connected to the second group were limited to two of the digestive enzymes (alpha-amylase and alkaline protease) and one metabolic enzyme (G6PDH), as well as members of the Proteobacteria phylum with respect to the assignation of the prevalent species. The latter was particularly noticeable in the case of the H50 treatment.

**Figure 5:**
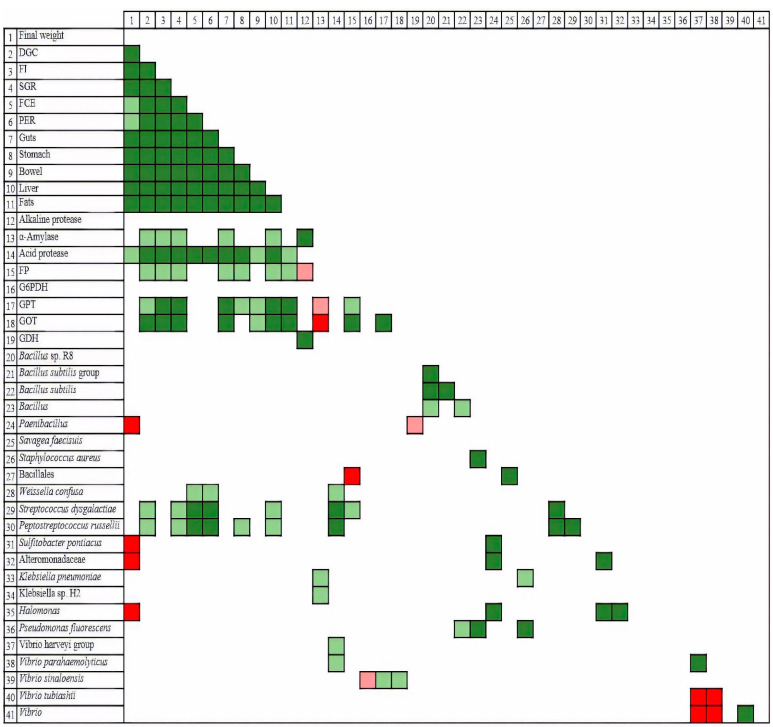
Correlations between parameters according to Pearson correlation analysis: positive results are depicted in green and negative results in red. Dark and light colors reveal significance at 99% and 95% confidence levels, respectively.

Specific connections between parameters were better reflected in the correlation analysis (Figure 5). According to the results, all biometric parameters were closely interrelated. Enzymes such as alpha-amylase, acid protease, FBPase, GPT and GDH also showed strong correlations with most of the biometric parameters. Regarding the microbial community, two species were relevant on account of their influence on growth and nutritive indices: *Streptococcus dysgalactiae* and *Peptostreptococcus russellii*. *Weisella confusa*, in contrast to these two bacteria, was positively correlated with two significant indices, as was the case with FCE and PER. However, *Sulfitobacter pontiacus*, *Halomonas* and Altermononadaceae showed a strong negative correlation with the final weight.

## 4. Discussion

In recent years, the replacement of fishmeal in aquafeed has been studied widely. Specifically, in feed for *S. aurata*, the level of inclusion of HI or TM meal without compromising growth has been established as being over 25–30% [11,38]. Higher replacement percentages need to be studied thoroughly to deepen knowledge regarding the digestive and metabolic responses of fish to insect meal ingestion. In this experiment, diets with high levels of substitution of fishmeal with insect meal (50:50) were studied, as was HI meal with a modified fatty acid profile according to the method described by Fabrikov et al. [16].

Nutritional and growth indices (Table 4) showed better results for the control diet in all the indices determined, followed by T50. Piccolo et al. [38] obtained similar growth rates for *S. aurata* fed with a fishmeal-based diet and a diet including a 25% replacement with TM meal, which was lower than that in our experiment. Pulido-Rodriguez et al. [12] and Randazzo et al. [13] also obtained similar results with inclusion of levels of 32.4%. Other than different percentages of fishmeal substitution, these discrepancies might be related to varying specific growth rates, which were around 2 in our experiment versus values closer to 0.5 in Piccolo et al. [35] and to 1.5 in Pulido-Rodriguez et al. [12] and Randazzo et al. [13]; these values are in accordance with the different initial weights used, i.e., 100 g and 48.8 g, respectively, versus 7 g in our experiment. FCE and PER also worsened in the T50 diet in comparison to C treatment, whereas no differences were observed in the study by Piccolo et al. [38] at a replacement level of 25%.

Fish fed with HI meal, no matter the level of replacement, showed worse nutritional and growth indices, which agrees with results obtained by Karapanagiotidis et al. [11]. Nevertheless, values observed for FCR and PER in the cases of H30 and H50M diets seem to be slightly better than those from H50. In the case of H50M, this result might be a consequence of the improved fatty acids profile of HI; however, in the case of H30, it could be due to the lower level of insect inclusion. The influence of insect meal on growth can also be illustrated with morphometric indices (Table 4). Results obtained for these indices confirmed what the nutritional parameters pointed out, with HI diets producing lower values. However, no differences between C and T50 treatments were found in this case, which support the idea of the better nutritive quality of TM meal in comparison with HI for *S. aurata* juveniles, especially when the latter does not improve nutritional profile.

Data regarding HI substitution levels higher than 30% in *S. aurata* feeding are scarce, if non-existent, although some studies have been published with other species as subjects. Thus, Reyes et al. [39] described similar results for *Dicentrarchus labrax*, reporting a better acceptance of diets containing a 30% inclusion in comparison to those with a 50% replacement. In contrast, no differences in nutritional and growth indices were detected for growth responses between fish fed with diets including 30% HI meal or 50% TM meal, showing a slightly better nutritive utilization of HI by *D. labrax* than by *S. aurata*.

The response of fish to specific diets is dependent on digestibility, which, in turn, is primarily associated with protein assimilation [38,40]. As such, the differences in growth and nutritional indices between HI- and TM-based diets could be due to the different digestibility of feeds obtained for *S. aurata* (CDA H30: 79.13; CDA H50: 79.16; CDA T50: 93.20). These values of digestibility are in agreement with those obtained in *D. labrax* for HI [6] and higher than those obtained in TM [41]. The different digestibility in these two insect species contrast with those reported by Marono et al. [42], who obtained an in vitro crude protein digestibility lower than in the present study but that was similar among different samples of HI and TM. Chitin content could also affect digestibility, and TM meal contains less chitin than HI meal (Table 1), which may partly explain the different digestibility indices measured in each case. The high CDA found for H50M (CDA H50M: 91.57) might be attributable to microbiological factors, because chitin concentrations must be similar. According to the results, H50M-fed fish showed the most similar microbiome to those undergoing C treatment, and both were different from the rest of the diets. Such differentiation was mostly based on the higher population of members of the genus *Bacillus*, especially, to those belonging to the *Bacillus subtilis* group, whose chitinolytic properties are well recognized [43].

On the other hand, digestibility is also related to digestive enzyme activities. In this respect, the results indicate that insect meals induced changes in digestive enzyme activities, decreasing the activity of acid protease and increasing alkaline protease and alpha-amylase, especially for H50 and H50M. In trout fed with HI or TM meal at 15% or 30% fishmeal replacement, an increase in alkaline protease activity was also observed, although no affectation of acid protease activity was detected [44]. Conversely, decreases in alkaline protease and trypsin activities were described in *Argyrosomus regius* fed with a diet with 30% fishmeal replacement by TM [45], whereas the inclusion of HI in *D. labrax* feed did not affect intestinal protease activities [6]. The digestibility of protein from insects can be highly variable, depending on the proportion of chitin [40]. Coutinho et al. [45] explain these changes as a slowdown in intestinal transit due to chitin that causes decreases in digestive enzyme activities. Nevertheless, protein digestibility is mostly influenced by the amino acid contents in ADF fractions [46]. Hence, changes in digestive protease activities could be associated with the skleroprotein of an insect’s exoskeleton, in addition to the chitin effect, as proposed by Coutinho et al. [45]. On the other hand, there is a clear connection between enzyme activity and dietary habits [47]; both may influence and be influenced by the intestinal microbiome [48]. Thus, the detection of different enzyme responses due to changes in feed and microbial communities should not be surprising.

Alpha-amylase activity showed a tendency to increase activity at high levels of insect inclusion. The results described by other authors vary among fish or insect species. For *D. labrax* fed with a 45% replacement with HI [6], or *Argyrosomus regius* fed with a diet with 30% fishmeal replacement with TM [45], no changes in alpha-amylase activity were detected; however, in *Oreochromis mossambicus* and *Clarias gariepinus* fed with *Imbrasia belina*, an increased amylase activity was found [49]. In rainbow trout *(Oncorhychus mykiss*), the inclusion of 30% HI meal produced a reduction in alpha-amylase activity, whereas no effect was observed when TM meal was used. Reasons for such varying responses are not clear, especially due to the absence of data dealing with 1,4-α-glucose polymer content, i.e., the substrate for this enzyme.

On the other hand, the inclusion of insect meal as an ingredient in diets did not seem to alter the intermediary metabolism of glucose or amino acids, because none of the analyzed enzymes involved in such processes showed significant changes in comparison to the C diet. Similar results were found for rainbow trout [44] and sea trout (*Salmo trutta* m. *trutta*) at low levels of replacement of fishmeal with TM meal [50]. Insect species induced changes in the Vmax of GPT between HI treatments regarding T50. Fabrikov et al. [5] studied the adaptation of amino acid catabolism enzymes to HI or TM meal inclusion in the diet among different species, including *S. aurata*. They found that Vmax exhibited an increase no matter the insect used for the formulation of the diet. However, in the present study, results showed different trends related to different insect species. Thus, although TM replicated the results from Fabrikov et al. [5], i.e., the Vmax of GPT increased in value, HI tended to reduce activity. In the case of GOT, Fabrikov et al. [5] did not find changes in the Vmax when HI meal was included in the diet, whereas an increase was observed when fishmeal was partially substituted by TM meal. These different responses could be attributed to the inclusion level—15% and 30% replacements in the experiment by Fabrikov et al. [5] and 50% in this assay—which might have led to a reduction in feed intake in HI groups. This fact, as well as lower digestibility, potentially reduces the availability of amino acids to be deaminated and, consequently, decreases GOT activity. In T50-fed fish, feed intake is higher than in HI groups, with the subsequent increase in amino acid availability being utilized as energy or for gluconeogenesis purposes, promoting both transaminase activities (GPT and GOT).

### 4.1. Gut Microbiome Response to Dietary Insect Inclusion

Previous studies have reported variations in the gastrointestinal microbiota of fish due to modifications in their diets [51]. In this case, the shaping of the microbiota is a logical response of the gut environment to variations in the nutritional composition of the diet, given the plasticity of bacterial communities and their rapid adaptation to new conditions [21]. In relation to the partial replacement of fishmeal with insect meal, some studies described an increase in the diversity of the intestinal microbiome [52], although opposite results have been obtained depending on insect and fish species, insect life-cycle stage or replacement level [53,54]. In the present work, different responses seem to be preferably related to the level of replacement rather than insect species, as revealed by the β diversity. Curiously, greater differences with regard to C diet were detected at the lowest level of inclusion, H30, which may seem contradictory, although this result has previously been reported [53]. Microbial community structures are dependent on several factors, and the characteristics of diet is just one of them. Environmental, phylogenetic and physiological interactions come in a wide range of conditions that, in turn, can create particular habitats which affect and modulate the composition of the gut microbiome [55]. In this sense, insect meal shows properties that can affect its acceptance by fish [56], as is the case of chitin content and fat profile. Regarding the latter, HI is characterized by a high saturated fatty acid content, as well as an unbalanced fatty acid profile [54] that can negatively affect fish performance. Therefore, it is common practice to provide defatted or fatty-modified meals with the potential to reduce undesirable effects and improve meal composition [16]. Concerning the gut microbiome, our results seem to suggest that such treatments may promote a nutritional profile more akin to that of fishmeal, which caused a lower impact in bacterial communities in the intestinal tract. Therefore, these two diets, C and H50M, clustered together. The almost complete lack of studies comparing the effect of full-fat and defatted insect meal on the fish gut microbiome has made it difficult to validate such a hypothesis, although it has been described in relation to other species [57]. Moreover, several documents point out the higher nutritional quality and digestibility of meals with a modified fat profile [58].

The dominance of *Vibrio* and *Bacillus* representatives in the fish gut microbiome is a common reality [19]. Members of the *V. harveyi* group, which is dominant in the C/H50M cluster, are associated with pathogenic processes [59]. Thus, the handover from this group to *Vibrio* sp. in the H30/H50/T50 cluster can be considered as positive, because species other than pathogens have been described in the genus, as in the case of *V. alginolyticus* [60], which has been postulated as a probiotic in aquaculture. The second largest microbial group was the *Bacillus* genus and its relatives. The importance of these bacteria relies on their enzyme activity and, to a lesser extent, their immunostimulant ability and antimicrobial production [61]. Taking into account their beneficial activities, and their presence in all treatments, it is clear that they have a positive impact on fish performance and wellbeing.

Apart from these dominant groups, other species were differentially present in the intestinal tract because of insect meal replacement. Bacteria such as *Streptococcus dysgalactiae*, *Peptostreptococcus russellii*, and especially *Weisella confusa*, reached relatively important population densities in the C samples, although they were practically absent in the rest of the treatments. *W. confusa* is a lactic acid bacterium which is associated with some infectious processes in human beings [62], but not in fish [63]. Moreover, it has been postulated as a probiotic for some species in aquaculture [64]. *P. russellii* has been positively associated with intestinal homeostasis in different species [65], although there is no literature about this in relation to fish. In contrast, *S. dysgalactiae* have been described as pathogenic for both human and fish [66]. However, *Sulfitobacter pontiacus* and members of the family Alteromonadaceae, absent in C-fed fishes, were detected in insect meal samples, especially H30. The first is a recognized sulfite and thiosulfate-oxidizing bacterium that might promote organic matter degradation in aquatic environments [67], whereas the latter comprises both pathogenic and potentially probiotic species [68,69].

In short, the replacement of fishmeal with insect meal seems to promote a redistribution of the most dominant bacterial populations, Vibrionaceae and Bacillaceae, whereas it modifies the identity of minor species. Nevertheless, and taking into account the diversity of conditions in which assays were performed, more information dealing with the impact of dietary modifications on intestinal bacterial communities is needed to attain a better understanding of the particular factors that rule the microbial response.

### 4.2. Gut Microbiome/Fish Performance Correlations

Gut microorganisms play different roles with respect to host performance and health. Beneficial bacteria can excrete enzymes that contribute to assimilating nutrients, reducing the availability of attachment sites for pathogens and synthesizing antimicrobial compounds [18]. The so-called persistent microbiota, which comprise those bacteria residing permanently in the gut, are considered as main protagonists of the symbiotic relation between the gut microbiome and a host [70]. Transient species, mostly associated with external environmental and nutritional factors, are thought to play some significant roles depending on their colonization ability [71]. Thus, species able to survive the conditions in the gut and adhere to intestinal mucosa may become permanent residents [72] and perform primary functionality [73]. The result described in this paper showing the grouping of T50 and C samples, and leaving treatment most microbiologically similar to C, H50M, out of this group, may be explained by this theory. Moreover, the evolutionary closeness between species in T50 and C, which shows the possibility of sharing metabolic properties, sustains such a hypothesis. The positive relationship between both diets and fish performance was evident by means of pooling with all the biometric parameters and most of the metabolic enzymes. Relevant microorganisms in the gut microbiome of these treatments, such as the *Bacillus subtilis* group, were also in this group, as well as *W. confusa*, *S. dysgalactiae* and *P. russellii*. These latter three were statistically correlated with many of the biometric parameters, such DGC, SGR, FCE and PER, reflecting their involvement in the beneficial utilization of a diet by fish. Thus far, none of them have been considered as key players in fish performance. *S. dysgalactiae* has even been associated with pathogenic processes [66]. Nevertheless, it has been reported that this bacterium could be a member of the persistent gut microbiome in fish, although its specific origin is not yet clear [74]. On the other hand, it has been referenced as a bacteriocin-producing bacteria [75], which is a desirable characteristic on account of its ability to prevent the growth of some other pathogens. For its part, *W. confusa*, which is also an antibacterial compound producer [76], has shown potential to positively impact fish growth performance, as well as stimulate the activity of some digestive enzymes [77]. Similarly, *P. russelli* has been associated with beneficial effects in terms of fish health and growth, especially regarding activities dealing with protein degradation [78], as elucidated in this paper.

## 5. Conclusions

The partial replacement of fishmeal with insect meal has proved to be an effective strategy, although the results depend on insect species and their characteristics, as well as the percentage of replacement. The impact of meal replacement was also different according to the nature of the parameters considered. Thus, growth, nutrition and enzymatic parameters seem to be more affected by the insect species used for producing the meal, whereas the composition of the microbial community associated with the gastrointestinal tract is likely to be mostly influenced by the level of replacement and the pretreatment of insects. Thus, *T. molitor* meal (T50) caused the lesser degree of impact on growth performance as indicated by biometric and enzyme parameters. On the other hand, modified *H. illucens* meal (H50M) was the only treatment in which, for the most part, the microbial community structure was maintained. In addition, the results seem to assign a relevant role to certain minority species (*Peptostreptococcus russellii*, *Streptococcus dysgalactiae* and *Weisella confusa*) not related to fish gut microbiota thus far. These results are not conclusive, because different species are approved for use in aquaculture feed and conditions in which insects are reared and meals are obtained differ between trials; however, this study contributes to the subject and establishes the most adequate conditions to promote insect meal-based feed utilization in fish.

## Figures and Tables

**Table 1:** Proximal composition of fresh insect meal.

g/100 g Fresh Matter	HI	Fish-Fed HI	TM
Fat (g/100 g)	25.6	27.8	27.0
Moisture (g/100 g)	8.0	8.7	5.0
Protein (g/100 g)	28.5	30.5	39.1
Ash (g/100 g)	9.75	8.66	3.42
Phosphorus (g/kg)	7.0	8.1	7.5
Calcium (g/kg)	35.2	27.0	0.93
Chitin (g/100 g)	7.5	7.7	5.9

HI: *Hermetia illucens*; TM: *Tenebrio molitor*.

**Table 2:** Aminoacidic profile of insect meal.

g/100 g Fresh Matter	HI	Fish-Fed HI	TM
Asp (aspartate)	2.92	2.84	3.71
Thr (threonine)	0.95	0.97	1.44
Ser (serine)	1.43	1.44	2.49
Glu (glutamate)	3.19	3.37	4.98
Pro (proline)	1.58	3.43	3.04
Gly (glycine)	1.84	2.02	2.87
Ala (alanine)	2.37	2.44	3.92
Val (valine)	1.42	1.46	2.32
Met (methionine)	0.47	0.41	0.57
Ile (isoleucine)	0.91	0.89	1.31
Leu (leucine)	1.86	1.83	2.96
Tyr (tyrosine)	2.23	2.05	4.47
Phe (phenylalanine)	2.16	1.81	3.07
His (histidine)	1.07	1.07	1.77
Lys (lysine)	1.94	1.87	2.49
Arg (arginine)	1.24	0.98	1.81

HI: *Hermetia illucens*; TM: *Tenebrio molitor*.

**Table 3:** Ingredients and proximal composition of elaborated diets used in experiment.

Ingredients (g/100 g Fresh Matter)	C	H30	H50	H50M	T50
Fishmeal	35.9	25.3	18	18	18
HI meal	0	10.9	18	0	0
Fish-fed HI meal	0	0	0	18	0
TM meal	0	0	0	0	18
Wheat gluten	10.5	13	15.4	15	11.9
Soy protein concentrate	15.5	17.5	18.3	18.3	17
Wheat meal	16.4	13.4	11.5	11.7	17
Soy lecithin	1.3	1	0.5	0.5	0.5
Fish oil	12.2	10.4	9.5	9.7	9
Vitamins and minerals	2	2	2	2	2
Guar gum	2	2	2	2	2
Hemoglobin powder	4	4	4	4	4
Methionine	0.2	0.5	0.5	0.4	0.5
Lysine		0.1	0.4	0.4	0.1
Total	100	100	100	100	100
Proximal Composition (g/100 g Fresh Matter)					
Crude protein	43.9	43.5	42.8	42.9	43.1
Crude fat	17.2	17.6	17.1	17.6	18.0
Fiber	-	-	-	-	-
Ash	7.4	6.9	6.4	6.3	6.1

HI: *Hermetia illucens*; TM: *Tenebrio molitor*.

**Table 4:** Nutritional and growth indices and percentage of organs regarding final body weight of *Sparus aurata* fed with five experimental diets.

	C	H30	H50	H50M	T50
Wf	21.16 ± 0.70 ^a^	14.00 ± 0.56 ^c^	13.8 ± 0.15 ^c^	14.90 ± 0.10 ^c^	19.13 ± 0.15 ^b^
DGC	2.48 ± 0.09 ^a^	1.43 ± 0.09 ^c^	1.40 ± 0.03 ^c^	1.58 ± 0.02 ^c^	2.21 ± 0.03 ^b^
FI	3.10 ± 0.02 ^a^	2.43 ± 0.01 ^b^	2.48 ± 0.09 ^b^	2.56 ± 0.12 ^b^	3.16 ± 0.09 ^a^
FCE	0.94 ± 0.02 ^a^	0.79 ± 0.04 ^bc^	0.76 ± 0.01 ^c^	0.81 ± 0.03 ^bc^	0.85 ± 0.01 ^b^
PER	2.21 ± 0.03 ^a^	1.85 ± 0.10 ^bc^	1.79 ± 0.03 ^c^	1.91 ± 0.04 ^bc^	2.00 ± 0.03 ^b^
%Viscera	9.95 ± 0.32 ^a^	6.88 ± 0.44 ^b^	6.71 ± 0.58 ^b^	7.83 ± 0.39 ^b^	10.29 ± 1.02 ^a^
%Stomach	1.31 ± 0.09 ^a^	0.94 ± 0.01 ^b^	0.98 ± 0.15 ^b^	0.99 ± 0.10 ^b^	1.16 ± 0.15 ^ab^
%Intestine	3.93 ± 0.51 ^a^	2.95 ± 0.03 ^bc^	2.81 ± 0.37 ^c^	3.28 ± 0.27 ^abc^	3.74 ± 0.01 ^ab^
%Liver	1.92 ± 0.19 ^a^	1.30 ± 0.11 ^b^	1.15 ± 0.10 ^b^	1.43 ± 0.12 ^b^	1.96 ± 0.21 ^a^
%Visceral fat	1.83 ± 0.24 ^a^	1.43 ± 0.48 ^b^	1.12 ± 0.14 ^b^	1.19 ± 0.06 ^b^	2.09 ± 0.30 ^a^

Means (±SE) of three replicate tanks (30 fish/tank). Wf, final body weight; Wi, initial body weight (average value = 7.10 g); DGC (daily growth coefficient) = [(Wf1/3–Wi1/3)/t] × 100); FI (feed intake) = (daily feed intake/average body weight) × 100; FCE (feed conversion efficiency) = wet weight gain/dry feed intake; PER (protein efficiency ratio) = wet weight gain/crude protein intake. Different letters indicate significant differences (*p* < 0.05) based on Tukey–Kramer HSD test.

**Table 5:** Proximal composition of raw muscle and apparent digestibility coefficient of diet protein from *Sparus aurata* fed with experimental diets. All results are expressed as percentages.

	Moisture	Fat	Protein	Ash	ADC_protein_
C	71.93 ± 0.67 ^a^	4.70 ± 0.55 ^ab^	19.63 ± 0.43 ^a^	1.88 ± 0.06 ^ab^	93.86 ± 0.26 ^b^
H30	73.93 ± 0.29 ^b^	3.66 ± 0.52 ^ab^	18.70 ± 0.61 ^a^	2.14 ± 0.06 ^b^	79.13 ± 1.64 ^a^
H50	73.66 ± 0.27 ^ab^	3.06 ± 0.12 ^a^	18.83 ± 0.22 ^a^	2.76 ± 0.04 ^c^	79.16 ± 0.25 ^a^
H50M	74.26 ± 0.12 ^b^	3.60 ± 0.35 ^ab^	19.16 ± 0.39 ^a^	2.57 ± 0.06 ^c^	91.57 ± 0.28 ^b^
T50	72.43 ± 0.51 ^ab^	5.56 ± 0.54 ^b^	18.30 ± 0.21 ^a^	1.77 ± 0.08 ^a^	93.20 ± 0.23 ^b^

Means (±SE) of three replicate tanks (30 fish/tank). ADC_protein_ (apparent digestibility coefficient of the protein). Different letters indicate significant differences (*p* < 0.05) based on Tukey-Kramer HSD tests.

**Table 6:** Number of OTUs and values for some α biodiversity indices observed for different treatments. Biodiversity indices are shown for the entire community, as well as for the community comprising those OTUs representing 0.5% of the relative abundance in at least one of the samples.

	C	H30	H50	H50M	T50
Total number of OTUs	281 ± 67	335 ± 60	270 ± 23	206 ± 22	308 ± 45
Shannon-Wiener richness index					
Entire population	2.59 ± 0.94	3.10 ± 1.55	2.39 ± 0.98	2.85 ± 1.13	2.16 ± 1.44
OTUs > 0.5%	2.15 ± 0.77	1.53 ± 0.92	2.53 ± 0.77	2.34 ± 0.63	2.06 ± 0.99
Simpson dominance index					
Entire population	0.66 ± 0.18	0.79 ± 0.30	0.70 ± 0.15	0.77 ± 0.31	0.49 ± 0.27
OTUs > 0.5%	0.63 ± 0.18	0.42 ± 0.25	0.76 ± 0.28	0.74 ± 0.14	0.68 ± 0.26

**Table 7:** β Diversity indices (Sørensen-Dice) observed for the entire (A) and restricted communities (B). Qualitative results are depicted in the upper quadrant and quantitative in the lower quadrant.

(A)	C	H30	H50	H50M	T50	(B)	C	H30	H50	H50M	T50
C		0.45 ± 0.05	0.45 ± 0.06	0.48 ± 0.05	0.49 ± 0.09	C		0.89 ± 0.09	0.83 ± 0.07	0.89 ± 0.10	0.89 ± 0.09
H30	0.27 ± 0.12		0.42 ± 0.07	0.49 ± 0.02	0.60 ± 0.05	H30	0.27 ± 0.11		0.80 ± 0.42	0.97 ± 0.36	0.92 ± 0.33
H50	0.50 ± 0.25	0.56 ± 0.26		0.42 ± 0.05	0.49 ± 0.07	H50	0.52 ± 0.30	0.58 ± 0.33		0.78 ± 0.08	0.83 ± 0.07
H50M	0.64 ± 0.25	0.39 ± 0.28	0.59 ± 0.25		0.51 ± 0.04	H50M	0.65 ± 0.14	0.40 ± 0.27	0.61 ± 0.26		0.89 ± 0.10
T50	0.56 ± 0.25	0.67 ± 0.30	0.79 ± 0.23	0.60 ± 0.21		T50	0.56 ± 0.17	0.69 ± 0.34	0.80 ± 0.23	0.61 ± 0.21	

## Data Availability

Data have been deposited in the Institutional Repository of the University of Almería.
